# Pterostilbene, an active constituent of blueberries, enhances innate immune activation and restricts enterovirus D68 infection

**DOI:** 10.3389/fimmu.2023.1118933

**Published:** 2023-02-09

**Authors:** Qingran Yang, Huili Li, Zhaoxue Li, Jiaxin Yang, Zhe Zhang, Lili Zhang, Haoran Guo, Wei Wei

**Affiliations:** ^1^ Key Laboratory of Organ Regeneration and Transplantation of Ministry of Education, Institute of Translational Medicine, First Hospital, Jilin University, Changchun, Jilin, China; ^2^ Institute of Virology and Acquired Immune Deficiency Syndrome (AIDS) Research, First Hospital, Jilin University, Changchun, Jilin, China

**Keywords:** EV-D68, pterostilbene, pinostilbene, interferon, innate immunity, antivirals

## Abstract

Enterovirus D68 (EV-D68) is a globally re-emerging respiratory pathogen implicated in outbreaks of severe respiratory illnesses and associated with acute flaccid myelitis. However, effective vaccines or treatments for EV-D68 infections remain scarce. We demonstrated that the active constituent of blueberries, pterostilbene (Pte), and its major metabolite, pinostilbene (Pin), facilitated innate immune responses in EV-D68-infected human respiratory cells. Pte and Pin treatment clearly relieved EV-D68-triggered cytopathic effects. Importantly, both Pte and Pin disrupted viral RNA replication (EC_50_ rank from 1.336 to 4.997 µM) and infectious virion production in a dose-dependent manner, without cytotoxicity at virucidal concentrations. Pte- or Pin-treated respiratory cells did not show any influences on EV-D68 entry but showed substantially decreased viral RNA replication and protein synthesis. Finally, we showed that Pte and Pin broadly suppressed the replication capacity of circulating EV-D68 strains isolated from recent pandemics. In summary, our results suggest that Pte and its derivative, Pin, enhance host immune recognition of EV-D68 and suppress EV-D68 replication, which represents a promising strategy for antiviral drug development.

## 1 Introduction

Enterovirus D68 (EV-D68), a member of the genus *Enterovirus*, belongs to the family *Picornaviridae* (1). EV-D68 contains a single positive-stranded RNA genome that includes an open reading frame (ORF). The ORF encodes the viral polyprotein, which can be digested by viral proteases to generate structural proteins (VP1–4) and non-structural proteins (2A–C and 3A–D) (2). The EV-D68 particle consists of 60 copies of each of the subunit proteins, VP1, VP2, VP3, and VP4. Each of the 12 pentameric vertices circles a deep surface depression, which contributes to receptor binding (3). Previous studies have found that sialic acid on the host cytomembrane facilitates EV-D68 infection (4). Human intercellular adhesion molecule 5 has been identified as a virus receptor for sialic acid-dependent and -independent EV-D68 viruses (5).

Contrary to the majority of enteroviruses transmitted *via* the ‘fecal-oral’ route, EV-D68 is a respiratory virus, and its infection can cause acute respiratory symptoms. EV-D68 is more sensitive to low pH and prefers cold conditions (6). Recently, accumulating evidence has suggested that EV-D68 infection is associated with clusters of acute flaccid myelitis (7). EV-D68 was considered a rare virus for a long time after its initial identification in 1962 (8), until its largest outbreak in North America in 2014 (9). The prevalence of this virus has been increasing in recent years, although an effective anti-EV-D68 treatment remains unavailable. Therefore, there is an urgent need for antiviral agents to control EV-D68 infection and transmission.

Pterostilbene (Pte), a naturally abundant compound in blueberries, is a methoxylated analog of resveratrol (10). Pte has been extensively investigated because of its therapeutic advantages in decreasing insulin resistance; controlling glucose and lipid levels; relieving cardiovascular diseases, inflammatory diseases, and aging; and improving memory and cognition (11). Pte treatment facilitates apoptosis, cell cycle arrest, DNA damage, and overloaded autophagy in carcinoma cells without affecting normal cells, making it an ideal anti-tumor agent (12). However, whether Pte can serve as an antiviral drug against EV-D68 replication remains unknown.

Here, we demonstrated that treatment with Pte or its major metabolite, pinostilbene (Pin), activated host antiviral immunity in the presence of EV-D68 infection. This finding prompted us to investigate the effects of Pte and Pin on EV-D68 infection in human respiratory cells. The tested small-molecule inhibitors dramatically interfered with EV-D68-mediated cytotoxicity and suppressed viral RNA replication, protein synthesis, and infectious viral particle production. Therefore, our results demonstrate that Pte and Pin have potential clinical value in anti-EV-D68 therapy.

## 2 Materials and methods

### 2.1 Cells, virus, and reagents

A549 human lung adenocarcinoma cells (CCL-185, ATCC), human rhabdomyosarcoma (RD) cells (CCL-136, ATCC), and BEAS-2B human bronchial epithelial cells (CRL-9609, ATCC) were maintained in Dulbecco’s Modified Eagle medium (DMEM; Sigma-Aldrich, USA), supplemented with 10% fetal bovine serum (FBS; Biological Industries, USA) and 1% penicillin/streptomycin. All cells were cultured at 37°C and 5% CO_2_.

EV-D68 prototype Fermon (VR-1826, ATCC), isolated in California in 1962, and isolated EV-D68 circulating strains from the 2014 United States outbreak, US/MO/14-18947 (VR-1823D, ATCC) and US/KY/14-18953 (VR-1825D, ATCC), were propagated in RD cells at 37 °C, and stored at -80 °C. Viruses in the supernatants of infected cells were harvested three days post-infection following three freeze–thaw cycles. Samples were clarified by low-speed centrifugation and then passed through a 0.22 µm filter. Viral particles were pelleted using 20% sucrose and centrifuged at 28,000 rpm using an SW28 rotor for 120 min (Beckman). Purified virions were stored at −80 °C.

Pterostilbene (HY-N0828, 99.83% purity) and pinostilbene (HY-N3059, 98% purity) were purchased from MedChemExpress (Monmouth Junction, NJ, USA).

### 2.2 Cell viability assay

Approximately 1.6 × 10^4^ A549 or BEAS-2B cells per well were seeded into 96-well plates with complete media and incubated at 37 °C with 5% CO_2_. When the cells were 90% confluent, the cell culture medium was removed and replaced with dimethyl sulfoxide (DMSO) or dose-gradient pterostilbene/pinostilbene. Cells were incubated for 24 h, and then 10 µL of Cell Counting Kit-8 reagent (CCK-8; MCE, Cat No.: HY-K0301) was added to each well. Absorbance was detected after 30 min using a BioTek plate reader (BioTek Instruments, Inc., USA) at a wavelength of 450 nm. Each data point represents the average of three technical replicates. 50% cytotoxicity concentrations (CC_50_) of pterostilbene or pinostilbene were calculated.

### 2.3 RNA quantification using qRT-PCR

Total RNA was isolated from cells using TRIzol (Life Technologies) according to the manufacturer’s instructions and included the DNase I digestion step. RNA was then converted to cDNA using a reverse transcription kit (Transgen Biotech, Beijing, China). PCR amplification was carried out on a LightCycler 480 Instrument II (Roche, Basel, Switzerland) with SYBR Green Supermix (Monad Biotech Co., Ltd., Wuhan, China). PCR cycling conditions used were: 95 °C for 2 min, followed by 40 cycles of 95 °C for 30 s and 60 °C for 1 min, with a final dissociation step of 95 °C for 5 min. Single peaks in the melting curve analysis indicated specific amplicons. The qRT-PCR was performed using the following primer sequences: glyceraldehyde-3-phosphate dehydrogenase (GAPDH) forward primer, 5′-GCAAATTCCATGGCACCGT-3′; GAPDH reverse primer, 5′-TCGCCCCACTTGATTTTGG-3′; EV-D68 forward primer, 5′-TGTTCCCACGGTTGAAAACAA-3′; EV-D68 reverse primer, 5′-TGTCTAGCGTCTCATGGTTTTCAC-3′; interferon (IFN)-β forward primer, 5′-TTGTGCTTCTCCACTACAGC-3′; IFN- β reverse primer, 5′-CTGTAAGTCTGTTAATGAAG-3′; interferon-induced protein with tetratricopeptide repeats 1 (IFIT1) forward primer, 5′-CAACCAAGCAAATGTGAGGA-3′;IFIT1 reverse primer, 5′-AGGGGAAGCAAAGAAAATGG-3′; MX dynamin-like GTPase 1 (MX1) forward primer, 5′- GTTTCCGAAGTGGACATCGCA-3′; and MX1 reverse primer, 5′- CTGCACAGGTTGTTCTCAGC-3′. The relative levels of EV-D68 RNA in different samples were determined using the comparative 2^-△△CT^ method with normalization against the *GAPDH* gene (5).

### 2.4 Viral attachment assay

Cells were washed with cold (4 °C) or pre-warmed (37 °C) phosphate-buffered saline (PBS), and then incubated with EV-D68 (multiplicity of infection, MOI = 1). After incubation at 4 °C or 37 °C for 2 h, cells were washed with cold or pre-warmed PBS to remove unbound viruses. Total RNA was extracted and relative viral RNA level was determined using qRT-PCR, as mentioned above.

### 2.5 Virus titer assay

Viral titers were determined using an endpoint dilution assay (EPDA). Briefly, RD cells were cultured under standard conditions in 96-well plates at a density of 10,000 cells/well. EV-D68 was serially diluted (10-fold) in DMEM containing 1% FBS and added to the cells. Viral titers were determined by the appearance of cytopathic effects (CPEs) in RD cells using microtitration analysis in accordance with the Reed–Muench method (13).

### 2.6 Immunoblotting

Cell or supernatant samples were harvested and boiled in loading buffer (0.08 M Tris, pH 6.8, with 2.0% SDS, 10% glycerol, 0.1 M dithiothreitol [DTT], and 0.2% bromophenol blue), followed by separation on sodium dodecyl sulphate–polyacrylamide gel electrophoresis and transferred to nitrocellulose membranes using a semi-dry apparatus (Bio-Rad, USA). Membranes were incubated with primary antibodies (anti-EV-D68 VP1 polyclonal antibody [Genetex, GTX132312]; anti-β-actin monoclonal antibody [Sigma, A3853]). Standard alkaline phosphatase-conjugated anti-rabbit IgG or anti-mouse IgG secondary antibodies (goat anti-rabbit IgG [Jackson ImmunoResearch Laboratories, code:115-005-045]; goat anti-mouse IgG [Jackson ImmunoResearch Laboratories, code: 115-055-062]) were then used. The membranes were stained with 5-bromo-4-chloro-3-indolyl phosphate and nitrotetrazolium blue chloride (Sigma-Aldrich) and visualized for band quantification.

### 2.7 Enzyme-linked immunosorbent assay

The quantification of interferon-α (IFN-α) or interferon-β (IFN-β) in the cell culture media was detected by ELISA kit (Shanghai Jianglai Industrial Limited by Share Ltd., JL12191, JL19215, China) according to the manufacturer’s instructions. A monoclonal antibody specific for IFN-α or IFN-β has been coated onto the wells of the micotiter strips(96-well ELISA plate) provided. Samples, including standards of known IFN-α or IFN-β concentrations, control specimens or unknowns were pipetted in triplicate into these wells. During the first incubation, the standards or samples and a biotinylated monoclonal antibody specific for IFN-α or IFN-β were simultaneously incubated. After washing, the enzyme Streptavidin-HRP, that binded the biotinylated antibody was added, incubated and washed. A TMB substrate solution was added which acted on the bound enzyme to induce a colored reaction product. The intensity of this colored product was directly proportional to the concentration of IFN-α or IFN-β present in the samples.The absorbance value was measured at 450 nm wavelength using a BioTek ELISA Reader (BioTek Instruments, Inc., USA).

### 2.8 Lentivirus production and gene silencing

HEK293T cells were co-transfected with plasmids sh-IRF3-pLKO.1 or non-targeting shRNA as shcontrol plus pRSV-Rev (12253), pMDLg/pRRE (12251), and pCMV-VSV-G (8454) (Addgene) with PEI. 48h post transfection, supernatants containing packaged lentivirus were harvested and cleared by centrifugation. Then A549 cells were transduced with the lentiviruses for 2 days. Puromycin (1.5 µg/ml) was then added into the culture medium to screen for stable cell lines in which IRF3 or control was shut down. Knockdown efficiency of IRF3 gene was examined by immunoblotting.

### 2.9 Statistical analysis

All statistical analyses were performed using GraphPad Prism software (version 8.0). Statistical analyses were performed using one-way analysis of variance (ANOVA). Statistical significance was set at *P* < 0.05.

## 3 Results

### 3.1 Pte facilitates innate immune activation during EV-D68 infection

**Figure 1:**
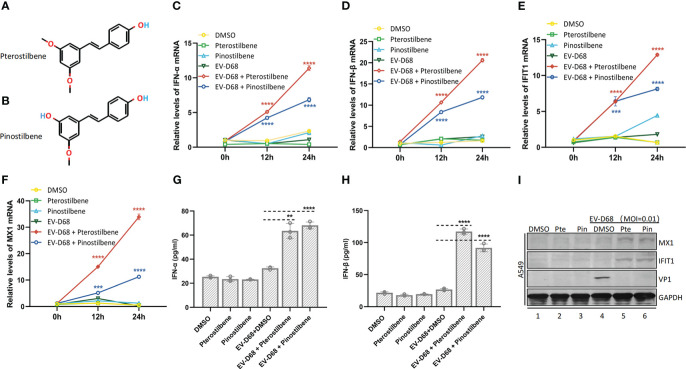
Pterostilbene (Pte) and pinostilbene (Pin) enhance EV-D68-induced innate immune responses. **(A)** Structure of Pte (molecular formula: C_16_H_16_O_3_). **(B)** Structure of Pin (molecular formula: C_15_H_14_O_3_). **(C–F)** Gene expression of *IFN-α* **(C)**, *IFN-β* **(D)**, *IFIT1* **(E)**, and *MX1* **(F)**, respectively, as indicators of virus-stimulated innate immune response. The mRNA was quantified by qRT-PCR at 0, 12, and 24 h post-infection. **(G, H)** Detection of IFN-α and IFN-β protein concentrations in supernatants by ELISA. **(I)** Detection of MX1 and IFIT1 in cells by Immunoblotting. Supernatants or cells were collected 24 h post-infection or mock-infection. Error bars indicate the standard deviation (**, *P* < 0.01; ***, *P* < 0.001; ****, *P* < 0.0001). EV-D68, Enterovirus D68; IFN, interferon; IFIT1, interferon-induced protein with tetratricopeptide repeats 1; MX1, MX dynamin-like GTPase 1; qRT-PCR, quantitative reverse transcription polymerase chainreaction.

Pterostilbene (trans-3,5-dimethoxy-4-hydroxystilbene) and pinostilbene (3,4-dihydroxy-5-methoxy-trans-stilbene), naturally occurring methylated derivatives of resveratrol (3,5,4-trihydroxystilbene), exert anti-inflammatory and immunomodulatory effects (**Figures 1A, B**). First, we investigated the effects of Pte and Pin on the host innate immune response to EV-D68 infection. A549 human lung adenocarcinoma cells were treated with 25 μM Pte or Pin in the presence or absence of EV-D68 (MOI = 0.01). Endogenous mRNA levels of the IFN-α, IFN-β and interferon-stimulated genes, *IFIT1* and *MX1*, were measured at 0, 12, and 24 h post-infection (**Figures 1C-F**). The protein expression of IFN-α, IFN-β, MX1 and IFIT1 in supernatant or cell was also detected (**Figures 1G-I**). The data indicated that single treatment with Pte or Pin during EV-D68 infection did not influence intrinsic innate immune status, as there were no observable changes in the expression of IFN-α, IFN-β, *IFIT1*, or *MX1* (**Figures 1C–I**). However, a dramatic increase in IFN-α and IFN-β expression was detected in EV-D68-infected cells in the presence of Pte or Pin in a time-dependent manner (**Figures 1C, D, G, H**). The changes in interferon-stimulated gene (ISG) expression levels further supported that Pte or its derivative, Pin, could enhance the innate immune response to EV-D68 infection, even though EV-D68 infection itself did not trigger immune activation (**Figures 1E, F, I**).

### 3.2 Pte and Pin inhibit EV-D68 replication

**Figure 2:**
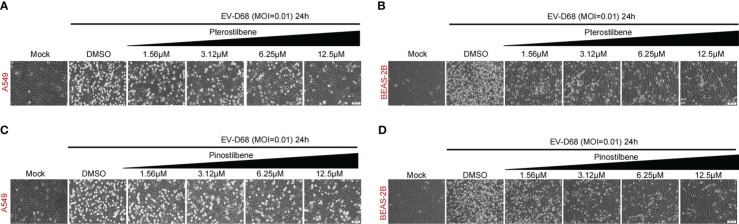
Pterostilbene (Pte) and pinostilbene (Pin) relieve CPEs in EV-D68-infected respiratory cells. **(A, B)** Indicated cells were pre-treated with increasing concentrations of Pte or DMSO vehicle before infection or mock-infection with EV-D68 virus. CPEs were observed 24 h post-infection. **(C, D)** Indicated cells were pre-treated with increasing concentrations of Pin or DMSO vehicle before infection or mock-infection with EV-D68 virus. CPEs were observed 24 h post-infection. EV-D68, Enterovirus D68; DMSO, dimethyl sulfoxide; CPE, cytopathic effects.

Research has demonstrated that type-I IFN potently inhibits EV-D68 infection (14). Therefore, we investigated the anti-EV-D68 activities of Pte and Pin. In vehicle-treated respiratory A549 and BEAS-2B cells, EV-D68 infection triggered clear CPEs. However, Pte and Pin treatment relieved virus-associated CPEs in a dose-dependent manner (**Figure 2**).

**Figure 3:**
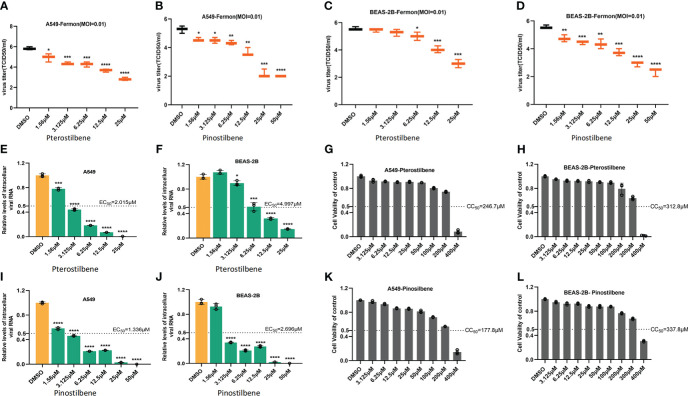
Pterostilbene (Pte) and pinostilbene (Pin) inhibit EV-D68 replication. **(A–D)** Determination of progeny virion production. Supernatants were collected 24 h post-infection and viral titers were determined by standard plaque assay. **(E–J)** qRT-PCR assessment of EV-D68 RNA replication. **(G–L)** Cell viability assay. Cellular toxicity was evaluated by CCK-8 assay and is expressed as the percentage relative to DMSO vehicle-treated control cells. Experiments were performed in triplicate. Error bars indicate the standard deviation (*, *P* < 0.05; **, *P* < 0.01; ***, *P* < 0.001; ****, *P* < 0.0001). EV-D68, Enterovirus D68; DMSO, dimethyl sulfoxide; qRT-PCR, quantitative reverse transcription polymerase chain reaction.

We also treated A549 cells with increasing concentrations of Pte or Pin (1.56, 3.125, 6.25, 12.5, and 25 μM), followed by challenge with EV-D68 prototype Fermon virus (MOI = 0.01). Pte and Pin treatment substantially decreased the viral titers of EV-D68 progeny virions compared to those of vehicle DMSO treatment (**Figures 3A, B**). Similar results were also obtained in BEAS-2B cells (**Figures 3C, D**). Furthermore, qRT-PCR data supported our conclusion by confirming that Pte and Pin suppressed EV-D68 RNA replication in A549 (**Figures 3E, I**) and BEAS-2B (**Figures 3F, J**) cells. The EC_50_ values of Pte against EV-D68 infection were 2.015 μM (A549 cells) and 4.997 μM (BEAS-2B cells). Pin showed more efficient anti-EV-D68 activity, with EC_50_ values of 1.336 μM (A549 cells) and 2.696 μM (BEAS-2B cells). Furthermore, the cell viability assay showed no substantial changes in cytotoxicity in cells treated with concentrations as high as 100 μM Pte or Pin (**Figures 3G–L**). The CC_50_ values of Pte were 246.7 μM (A549 cells) and 312.8 μM (BEAS-2B cells). For Pin, the CC_50_ values were 177.8 μM (A549 cells) and 337.8 μM (BEAS-2B cells). Selection index (SI) of antiviral effect = CC_50_/EC_50._ SI value of Pte in A549 cells was 122.4; SI value of Pin in A549 cells was 133.1; SI value of Pte in BEAS-2B cells was 62.6; SI value of Pin in BEAS-2B cells was 125.3.

### 3.3 Pte and Pin do not influence EV-D68 entry into cells

**Figure 4:**
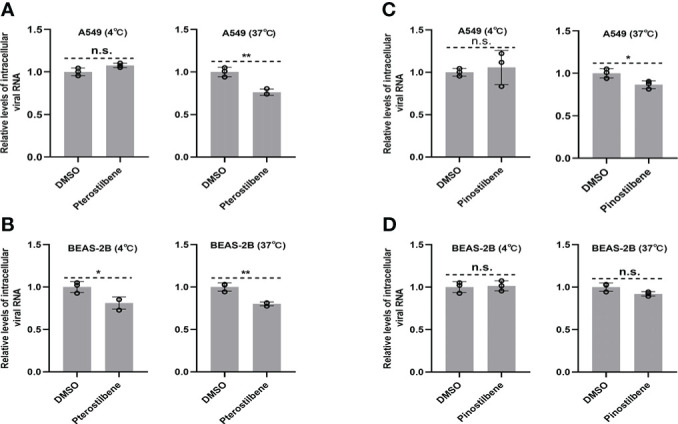
Pterostilbene (Pte) and pinostilbene (Pin) do not influence EV-D68 attachment or entry into cells. **(A–D)** Cells were treated with Pte, Pin, or DMSO vehicle in 12-well plates under standard conditions. Cells were incubated at 4°C or 37°C for 30 min before infection and then incubated with EV-D68 viruses for 2 h. The qRT-PCR was performed to quantify viral RNA. EV-D68, Enterovirus D68; DMSO, dimethyl sulfoxide; qRT-PCR, quantitative reverse transcription polymerase chain reaction. Error bars indicate the standard deviation (n.s., no significance, *, *P* < 0.05; **, *P* < 0.01).

Next, we investigated the effects of Pte and Pin on EV-D68 entry into host cells. Viral attachment and entry experiments were performed in the presence of 25 μM Pte, 25 μM Pin, or DMSO. Viral attachment (at 4 °C) and entry (at 37 °C) were assessed using the quantification of intracellular EV-D68 RNA 2 h post-infection. Repeated experiments confirmed that neither Pte nor Pin treatment obviously disrupted the attachment of EV-D68 to A549 (**Figures 4A, C**) or BEAS-2B cells (**Figures 4B, D**), although there were slight statistical differences seen in **Figures 4A-C**. Therefore, Pte/Pin inhibited EV-D68 infection during the post-entry step.

### 3.4 Pte and Pin inhibit viral RNA replication and VP1 synthesis

**Figure 5:**
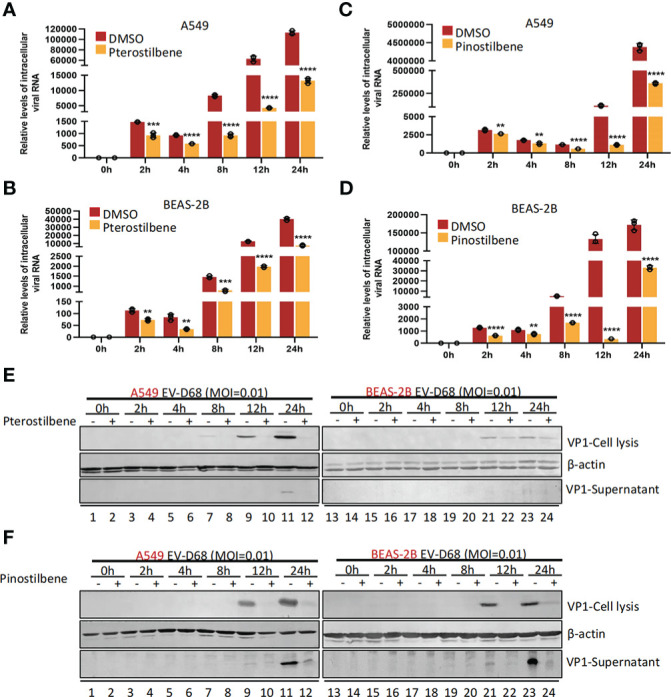
Pterostilbene (Pte) and pinostilbene (Pin) suppress EV-D68 RNA replication and VP1 synthesis post viral entry. A549 or BEAS-2B cells were pre-treated with Pte (25 µM), Pin (25 µM) or DMSO for 2 h and subsequently infected with EV-D68 at an MOI of 0.01. Cell or supernatant samples were collected at 0, 2, 4, 8, and 12 h post-infection. Relative intracellular viral RNA was detected using qRT-PCR **(A–D)**, Viral structural protein VP1 was measured by immunoblotting **(E, F)**. EV-D68, Enterovirus D68; DMSO, dimethyl sulfoxide; qRT-PCR, quantitative reverse transcription polymerase chain reaction; MOI, multiplicity of infection. Error bars indicate the standard deviation (**, *P* < 0.01; ***, *P* < 0.001; ****, *P* < 0.0001).

We next sought to determine the part of the EV-D68 life cycle after entry that was inhibited by Pte and Pin treatment. We analyzed EV-D68 growth curves during infection of A549 (MOI = 0.01) and BEAS-2B cells (MOI = 0.01) in the presence of 25 μM Pte, 25 μM Pin, or 0.1% DMSO (**Figures 5A–D**). Viral RNA was quantified at the indicated times after viral infection using qRT-PCR. We noted that Pte and Pin treatment substantially decreased EV-D68 RNA compared to that in the vehicle control at 2 h post-infection (**Figures 5A–D**). Consistent with these results, we observed a substantial reduction in EV-D68 VP1 protein accumulation in the intracellular portion and supernatant of Pte/Pin-treated cells compared to that of vehicle-treated cells (**Figures 5E, F**). Hence, treatment with Pte and Pin serves to impede the early stages of EV-D68 infection.

### 3.5 Pte and Pin suppress isolated circulating strains of EV-D68

**Figure 6:**
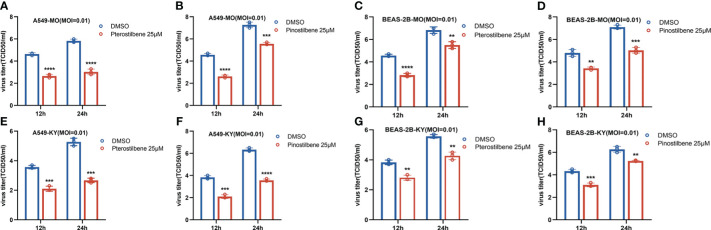
Pterostilbene (Pte) and pinostilbene (Pin) inhibit isolated circulating strains of EV-D68. **(A–H)** A549 or BEAS-2B cells were pre-treated with 25 μM Pte, or 25 μM Pin, or DMSO vehicle and then infected with US/MO/14-18947 (MO) or US/KY/14-18953 (KY). Cells were incubated under standard conditions. The titers of progeny virions were determined 12 or 24 h post-infection. The experiment was performed thrice. Error bars indicate the standard deviation (**, *P* < 0.01; ***, *P* < 0.001; ****, *P* < 0.0001). EV-D68, Enterovirus D68; DMSO, dimethyl sulfoxide; MO and KY, isolated EV-D68 circulating strains.

Accumulating evidence has demonstrated distinct features between the EV-D68 prototype virus and circulating strains (15). We measured the broad-spectrum activity of Pte and Pin against primary EV-D68 isolates (US/MO/14-18947 [MO] and US/KY/14-18953 [KY]). Similar to the inhibition of EV-D68 prototype Fermon, Pte and Pin treatment substantially decreased the viral titers of the MO (**Figures 6A–D**) and KY strains (**Figures 6E–H**) compared to the vehicle control treatment in A549 and BESA-2B cells. In summary, Pte and its derivative, Pin, exert broad antiviral effects against both the prototype and circulating EV-D68 viruses.

### 3.6 Pte and Pin mediate innate immune activation through the IRF3 signaling pathway

**Figure 7:**
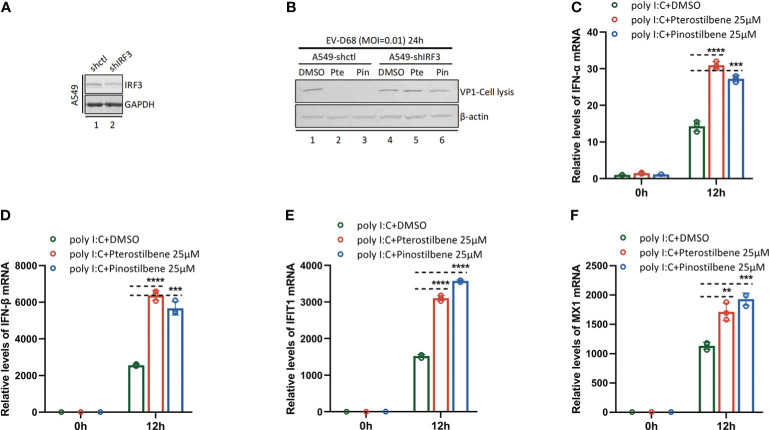
Pterostilbene (Pte) and pinostilbene (Pin) mediate innate immune activation through IRF3 signaling pathway. **(A)** IRF3 gene knock-down efficiency validation by Immunoblotting. **(B)** A549-shctl or A549-shIRF3 cells were pre-treated with Pte (25 µM), Pin (25 µM) or 0.1% DMSO for 2h and then infected with EV-D68 at an MOI of 0.01. Cells were collected 24 h post-infection. Viral protein VP1 was detected using Immunoblotting. **(C–F)**, A549 cells were pre-treated with Pte, Pin or DMSO for 2h, then transfected with Poly I:C (1 µg per well). Cells were collected at 0h and 12h post-transfection. Relative intracellular IFN-α, IFN-β, MX1 and IFIT1 mRNA were detected using qRT-PCR. Error bars indicate the standard deviation (**, *P* < 0.01; ***, *P* < 0.001; ****, *P* < 0.0001).

We further proved that Pte and Pin activate innate immunity through the IRF3 signaling pathway. Synthesis of viral protein VP1 was significantly attenuated in A549-shIRF3 cell lines (Gene knock-down efficiency validation: **Figure 7A**) in the presence of Pte (25 μM) or Pin (25 μM) (**Figure 7B**). It suggested that Pte and Pin mediated innate immune activation through the IRF3 signaling pathway and achieved antiviral effects. In addition, Pte and Pin could enhance the innate immune activation effect by the RNA virus mimic Poly I:C (**Figures 7C-F**). This indicated that Pte and Pin may play a role in the interplay between viral RNA replication and activation of the host immune system.

## 4 Discussion

During the recent coronavirus pandemic, the transmission of other respiratory pathogens worldwide, including EV-D68, dramatically decreased. However, by 2022, several EV-D68 cases have been reported, which may be due to the gradual recovery of social activities (16). Recent research advances in EV-D68 pathogenesis further support that EV-D68 is a neurotropic virus, and its infection is associated with a higher risk of acute flaccid myelitis (17–19). Thus, there is an urgent need to identify safer and more effective anti-EV-D68 drugs. In this study, we showed that natural products extracted from blueberries (Pte and its derivative, Pin) are potent inhibitors of EV-D68 replication without cytotoxicity at virucidal concentrations.

Host innate immune defense is critical in controlling the invasion of foreign pathogens at an early stage after they enter the body (20). Viruses have evolved diverse strategies to disrupt or evade intracellular innate immune recognition (21, 22). Previous studies have demonstrated that viral proteins encoded by EV-D68, such as 2A, 3C, and 3D, inhibit RIG-I-like receptors, Toll-like receptor-dependent innate immune activation, and IFN production (14, 23–28). Studies have shown that treatment with exogenous IFN robustly blocks EV-D68 infection in cell culture systems (14). Although we did not observe innate immune activation in EV-D68-infected immortalized cells, EV-D68 infection can initiate cell-type-specific antiviral signaling in human airway epithelial cells and enteroids, which contributes to EV-D68 control (29). Hence, boosting host innate immune recognition and EV-D68 activation is an optimal strategy for drug development. Our data showed that Pte and Pin substantially increased the expression of IFN-I and IFN-stimulated genes in EV-D68-infected cells. Enhanced innate immune activation by Pte/Pin stimulates IFN signaling to mobilize host antiviral factors and clear viral infections. During the preparation of our manuscript, a new study reported that Pte could inhibit influenza virus infection by modulating innate immune responses (30), which also supports our conclusion. Interestingly, we noted that treatment with Pte and Pin did not influence the innate immune status in human respiratory cells in the absence of EV-D68, which highlights the safety of Pte and Pin for future clinical use.

Recent studies have identified a series of IFN-stimulated genes that maintain anti-enterovirus activities that can block viral replication at different stages of the viral life cycle (31–33). We analyzed the effects of Pte/Pin on the entry of EV-D68 virions and growth curves of EV-D68 RNA replication and VP1 synthesis during infection. Both Pte and Pin dramatically interfere with EV-D68 RNA copy numbers and viral protein accumulation, although no influence was observed on viral invasion of cells. These data suggest that immune activation by Pte/Pin inhibits EV-D68 infection at the post-entry stages of infection. RIG-I-IRF3 is the main upstream signaling pathway that activates interferon. We designed A549 cell lines with stable knockdown of the IRF3 gene, in which even with the presence of Pte or Pin, the anti-EV-D68-effect was significantly impaired, suggesting that Pte or Pin mediates host innate immune activation associated with EV-D68 infection *via* the IRF3 signaling pathway. Poly I:C is an artificial synthetic double-stranded RNA (dsRNA) analog, a molecular pattern associated with viral infection which acts as a potent interferon inducer. Our research confirmed that Pte and Pin could further enhance the activation of interferon by Poly I:C, suggesting that Pte and Pin may have an impact on the interplay between viral RNA replication and host innate immune activation. However, the detailed mechanisms by which Pte/Pin inhibit immune evasion by EV-D68 and promote the blockage of viral replication require further clarification.

Pte exerts a wide range of biological effects on human health, including antioxidative, anti-aging, antitumor, and anti-inflammatory effects (34). An important advantage of Pte over resveratrol is its higher bioavailability. Improved lipophilic properties and oral absorption, higher cellular uptake, and a longer half-life than resveratrol also strengthen its potential for future clinical application (35). Pin is a major metabolite of Pte and plays an important role in the anti-colon cancer effects elicited by orally administered Pte (36). However, the antiviral activities of Pte and Pin, particularly their anti-enteroviral effects, have rarely been investigated. The identification of Pte as a novel anti-EV-D68 agent should accelerate its entry into clinical trials to control the EV-D68 pandemic. The antiviral effects of Pte and Pin against virus infection *in vivo* still need deeper investigation in further studies.

In summary, Pte- and Pin induced time-dependent innate immune activation during EV-D68 infection in human respiratory cells. Concomitantly, the antiviral immune status restricted EV-D68, which failed to complete its life cycle and generate progeny virions. These findings elucidate a novel antiviral strategy to block viral innate immune evasion using natural small-molecule inhibitors. These compounds should be further explored in future clinical investigations.

## Data availability statement

The raw data supporting the conclusions of this article will be made available by the authors, without undue reservation.

## Author contributions

QY, HL, and ZL, performed the experiments. WW, QY, JY, ZZ, LZ, and HG analyzed the data. WW, HG, and LZ wrote the paper with help from all authors. W.W. directed the project. All authors contributed to the article and approved the submitted version.
